# Selective Detection of Target Volatile Organic Compounds in Contaminated Air Using Sensor Array with Machine Learning: Aging Notes and Mold Smells in Simulated Automobile Interior Contaminant Gases

**DOI:** 10.3390/s20092687

**Published:** 2020-05-08

**Authors:** Toshio Itoh, Yutaro Koyama, Woosuck Shin, Takafumi Akamatsu, Akihiro Tsuruta, Yoshitake Masuda, Kazuhisa Uchiyama

**Affiliations:** 1 National Institute of Advanced Industrial Science and Technology (AIST), Shimo-shidami, Moriyama-ku, Nagoya 463-8560, Japan; sonic.h.0715@gmail.com (Y.K.); w.shin@aist.go.jp (W.S.); t-akamatsu@aist.go.jp (T.A.); a.tsuruta@aist.go.jp (A.T.); masuda-y@aist.go.jp (Y.M.); 2 Department of Information Science and Technology, Aichi Prefectural University, 1522-3 Ibaragabasama, Nagakute 480-1198, Aichi, Japan; 3 DENSO Corporation, 1-1, Showa-cho, Kariya 448-8661, Aichi, Japan; kazuhisa_uchiyama@denso.co.jp

**Keywords:** semiconductive-type gas sensor, age-related body odor, fungi odor, indoor-air contamination, machine learning, principal-component analysis (PCA), linear discriminant analysis (LDA)

## Abstract

We investigated the selective detection of target volatile organic compounds (VOCs) which are age-related body odors (namely, 2-nonenal, pelargonic acid, and diacetyl) and a fungal odor (namely, acetic acid) in the presence of interference VOCs from car interiors (namely, *n*-decane, and butyl acetate). We used eight semiconductive gas sensors as a sensor array; analyzing their signals using machine learning; principal-component analysis (PCA), and linear-discriminant analysis (LDA) as dimensionality-reduction methods; k-nearest-neighbor (kNN) classification to evaluate the accuracy of target-gas determination; and random forest and ReliefF feature selections to choose appropriate sensors from our sensor array. PCA and LDA scores from the sensor responses to each target gas with contaminant gases were generally within the area of each target gas; hence; discrimination between each target gas was nearly achieved. Random forest and ReliefF efficiently reduced the required number of sensors, and kNN verified the quality of target-gas discrimination by each sensor set.

## 1. Introduction

Every day, people access transportation in enclosed spaces such as cars, railways, and airplanes to commute to work or school and to travel. To ensure that these enclosed spaces form a comfortable environment, it is necessary to integrate measurement technology that allows for the objective evaluation of factors that lead to users’ pleasure or discomfort during transportation with analytical technology to instantaneously draw conclusions from the measurement results and apply appropriate feedback. When it becomes possible to quantify human sensation and automatically provide feedback, we can improve travel comfort, and therefore quality of life. For example, age-related body odors, the main components of which are *trans*-2-nonenal [1,2,3,4], pelargonic acid [5], and diacetyl [4,6], have distinctive smells. It may be difficult for the person emitting the smells to notice them, and other people in the area may be hesitant to inform someone about the unpleasantness of their odor and be forced to endure it. Some people worry excessively whether they smell unpleasant. In addition, although air conditioning is a tool for maintaining the comfort of an enclosed space, it is likely to generate fungi, such as *Cladosporium*, which diffuse unpleasant smells such as acids [7]; not only do fungi give off unpleasant odors, they may also affect health [8,9]. The problem in developing odor-sensing technologies is that enclosed spaces also diffuse other volatile organic compounds (VOCs) diffusing from the interior, such as *n*-decane and butyl acetate, which interfere with the detection of unpleasant smells. 

In this study, we measured simulated unpleasant fungus and age-related body odors using a sensor array in the presence of simulated contaminant VOCs from an automobile interior. Acetic acid, *trans*-2-nonenal, pelargonic acid, and diacetyl were used as simulated odors, and butyl acetate and *n*-decane were used as simulated contaminants because they were main components of odors and contaminants. Using these components, we investigated the possibility of discriminating fungus and age-related body odors in automobile interiors. Since each odor is composed of various components, it is desirable to study using mixed gases as each simulated odor. Therefore, we subsequently clarify the components of each odor with analyzing instruments, such as GC/MS. In this study, we used general semiconductor gas sensors [10,11], including commercially available sensors, as well as Pt-, Pd-, and Au-loaded SnO_2_, and bulk-response Ce_1−x_Zr_x_O_2_ sensors [12,13,14] as the sensor array. Pristine SnO_2_ possesses high sensitivity to oxyhydrocarbons [15]. Moreover, loading Pt, Pd, and Au to SnO_2_ can improve its sensitivity to aliphatic, halogenated, and aromatic hydrocarbons [16], so these sensors are suitable for detecting various VOCs. Ce_1−x_Zr_x_O_2_ possesses a high oxygen-diffusion coefficient to provide lattice oxygen for oxidizing VOCs, ensuring that the sensor is nearly unaffected by humidity. Therefore, adding Pt-, Pd-, and Au-loaded SnO_2_ and Ce_1−x_Zr_x_O_2_ to the sensor array can improve the array’s discrimination ability. [17]. Moreover, we investigated the application of various basic machine-learning methods to the sensor array for improving the discriminant ability. Previous research investigated sensor arrays using multiple gas sensors to be analyzed with various statistical methods, i.e., machine learning [18,19,20,21,22,23,24,25,26,27,28]. We also explored discriminating between several VOCs in a contaminant gas using a semiconductor sensor array with principal-component analysis (PCA) [17,29]. In manufacturing a measurement system, it is desirable to minimize the number of parts in the system. Moreover, when sensors with low discrimination ability are incorporated into a sensor array, inappropriate information is included, decreasing the discrimination accuracy of machine learning. Therefore, this study also examines the selection of appropriate sensors from the array to improve machine-learning accuracy.

## 2. Materials and Methods

### 2.1. Gas Sensors

We used four commercially available semiconductor metal oxide sensors (TGS 2600, 2602, 2610, and 2620; Figaro Engineering Inc., Minoh, Japan); two semiconductor 1 wt % Pt, 1 wt % Pd, and 1 wt % Au-loaded SnO_2_ sensors (#31b and #33b); and two semiconductor Zr-doped CeO_2_ sensors (nos. 9 and 71). The Pt-, Pd-, and Au-loaded SnO_2_ sensors, and the Zr-doped CeO_2_ sensors were prepared as in previous reports [14,30], and as summarized in another previous report [17]. Details of the sensors are summarized in Table 1.

### 2.2. Preparation of Target and Contaminant VOCs

**Figure 1:**
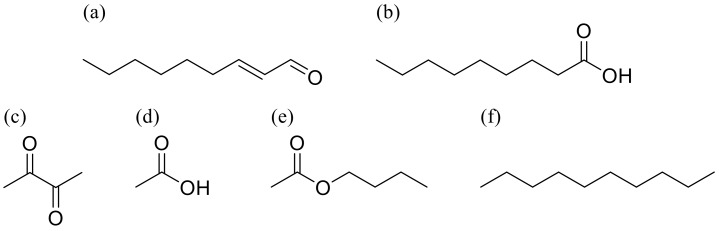
Structural formulas of target and contaminant gases. (**a**) *trans*-2-nonenal (2N); (**b**) pelargonic acid (nonanoic acid; PA), (**c**) diacetyl (2,3-butandione; DA), (**d**) acetic acid (AA), (**e**) butyl acetate (B), and (**f**) *n*-decane (D).

The molecular structures of the target VOCs (*trans*-2-nonenal, pelargonic acid (nonanoic acid), diacetyl (2,3-butandione), and acetic acid) and contaminant VOCs (butyl acetate and *n*-decane) are shown in Figure 1; *trans*-2-nonenal, pelargonic acid, and diacetyl are the main components of simulated age-related body odors [1–6]. Fungi in air-conditioning systems diffuse acids [7]. In this study, acetic acid was set as a target VOC on the basis of a result of our original analyzing study. Butyl acetate and *n*-decane are the main olfactory components from car interiors, also as analyzed by our original study. In this paper, *trans*-2-nonenal, pelargonic acid, diacetyl, acetic acid, butyl acetate, and *n*-decane are denoted as 2N, PA, DA, AA, B, and D, respectively.

**Figure 2:**
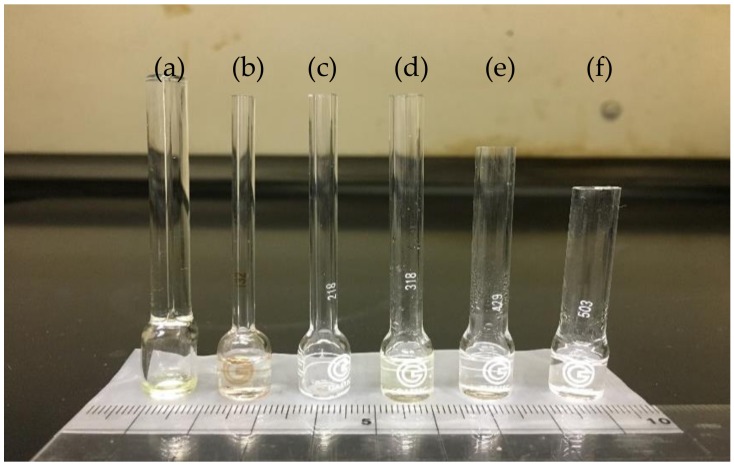
D-tubes for gas generator: (**a**) D-001, (**b**) D-01, (**c**) D-02, (**d**) D-03, (**e**) D-04, (**f**) D-05.

All target and contaminant gases were generated from their solvents by a Gastec Permeator PD-1B gas generator (Gastec, Ayase, Japan). The gas generator can be used with exclusive diffusion vessels, i.e., D-tubes, as shown in Figure 2. Liquids of the VOC sources were poured into the D-tubes, and a maximum of four D-tubes were placed into the diffusion chamber of the gas generator. The concentration of VOCs could be controlled by changing the kind of D-tube, chamber temperature, and the flow rate of the carrier gas. In this study, sources of all VOCs were placed into the same gas generator to generate mixed gases, so that the temperature and flow rate were kept at 35 °C and 200 mL/min, and the concentration was controlled by the kind of D-tube. Table 2 shows the combinations of D-tubes and their diffusing concentrations for the target and contaminant gases. Our analyses aimed to sense a single target gas with 0, 1, or 2 contaminant gases and two target gases with 0 or 1 contaminant gases. Table 3 shows the plot shape and size in PCA and linear discriminant analysis (LDA) scores (Figures 5, 7, 9 and 10) from each sensing analysis. 

### 2.3. Gas-Sensor Analysis

**Figure 3:**
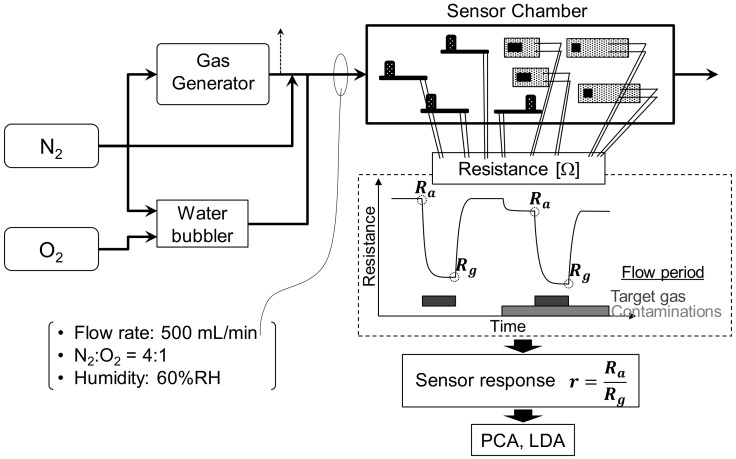
Schematic of flow apparatus for measuring sensor responses.

Sensor responses were measured using a flow apparatus as shown in Figure 3. To generate target and contaminant gases, 200 mL/min of nitrogen was introduced into the gas generator. To generate humid air, 200 mL/min of nitrogen and 100 mL/min of oxygen were introduced into a water bubbler. Additional dry nitrogen was added to adjust the total flow rate to 500 mL/min. Under these conditions, the N_2_/O_2_ ratio was maintained at 4, and relative humidity (RH) was 60%. The concentrations of target and contaminant gases are indicated in Table 3. Sensor response *r* is defined in Equation (1) as

(1) $$r=\frac{R_{a}}{R_{g}},$$

where *R_a_* is the electric resistance of sensor in humid pure air or in contaminants and *R_g_* is the resistance in the target gases.

### 2.4. Data Analysis

Discrimination of each target VOC was carried out using PCA and LDA [31], both of which are dimensionality-reduction methods. PCA and LDA find the axis that maximizes data variance and the separation between multiple classes, respectively. In this study, each class would be a different target gas. LDA is a supervised-learning method, whereas PCA is an unsupervised-learning method.

#### 2.4.1. PCA

PCA can be performed by solving the generalized eigenvalue problem in Equation (2):

(2) $$Av_{j}=\lambda_{j}v_{j},$$

where **A** is a matrix of correlation coefficients (Equation (3)):

(3) $$A=\left(\begin{array}{cccc} c_{11} & c_{12} & \cdots & c_{1n}\\ c_{21} & c_{22} & \cdots & c_{2n}\\ \vdots & \vdots & \ddots & \vdots \\ c_{n1} & c_{n2} & \cdots & c_{nn} \end{array}\right),$$

*c_ab_* is a correlation coefficient between sensors *a* and *b* (*c*_11_ = *c*_22_ = … = *c_nn_* = 1), *n* is the maximal dimension number (*i.e.*, number of sensors), **v_j_** (**v_1_**, **v_2_**, …, **v*_n_***) are eigenvectors, and *λ_j_* (*λ*_1_, *λ*_2_, …, *λ_n_*) are eigenvalues (*λ*_1_ > *λ*_2_ > … > *λ_n_*). Eigenvector **v_j_** is a matrix of *n* rows and 1 column (Equation (4)):

(4) $$v_{j}=\left(\begin{array}{c} v_{1j}\\ v_{2j}\\ \vdots \\ v_{nj} \end{array}\right).$$

Normalized scores obtained from sensor responses were converted into the PCA scores by the eigenvectors (Equation (5)):

(5) x′=xv,

where **x′** is a matrix of PCA scores, **x** is a matrix of normalized scores (Equation (6)):

(6) $$x=\left(\begin{array}{cccc} x_{11} & x_{12} & \cdots & x_{1n}\\ x_{21} & x_{22} & \cdots & x_{2n}\\ \vdots & \vdots & \ddots & \vdots \\ \vdots & \vdots & \ddots & \vdots \\ x_{N1} & x_{N2} & \cdots & x_{Nn} \end{array}\right),$$

and **v** is a matrix of eigenvectors (Equation (7)):

(7) $$v=\left(\begin{array}{cccc} v_{11} & v_{12} & \cdots & v_{1n}\\ v_{21} & v_{22} & \cdots & v_{2n}\\ \vdots & \vdots & \ddots & \vdots \\ v_{n1} & v_{n2} & \cdots & v_{nn} \end{array}\right).$$

Normalized scores *x_it_* were calculated according to Equation (8):

(8) $$x_{it}=\frac{\left(r_{it}-\bar{r_{t}}\right)}{\sigma_{t}},$$

where *t* is the sensor index (with a maximum of *n*), *i* is the sensor-response-analysis index (with a maximum of *N*), *r_it_* is the sensor-response value of sensor *t* (1, 2, …, *n*) on analysis *i* (1, 2, …, *N*), $\bar{r_{t}}$ is the average sensor response of sensor *t*, and $\sigma_{t}$ is the standard deviation of sensor *t*. In this study, PCA was carried out using Origin 2017 software (Origin Lab Corporation, Northampton, MA, USA).

#### 2.4.2. LDA

LDA can also be performed by solving the generalized eigenvalue problem in Equation (2). However, in this case, matrix **A** is a product of an inverse of a within-class covariance matrix and an interclass covariance matrix (Equation (9)):

(9) $$A=S_{W}^{-1}S_{B},$$

where **S_W_** is a within-class covariance matrix (Equation (10); *T* indicates a transposed matrix):

(10) $$S_{W}=\frac{1}{N}\sum_{k=1}^{C}\sum_{i=1}^{N_{k}}\left(x_{i}-m_{k}\right){\left(x_{i}-m_{k}\right)}^{T},$$

**S_B_** is an interclass covariance matrix (Equation (11)):

(11) $$S_{B}=\frac{1}{N}\sum_{k=1}^{C}N_{k}\left(m_{k}-m\right){\left(m_{k}-m\right)}^{T},$$

**x_i_** is a matrix of normalized scores (Equation (12)):

(12) $$x_{i}=\left(\begin{array}{c} x_{i1}\\ x_{i2}\\ \vdots \\ x_{in} \end{array}\right),$$

**m** is a mean vector (Equation (13)):

(13) $$m=\frac{1}{N}\sum_{i=1}^{N}x_{i},$$

**m_k_** is a class mean vector of class *k* (Equation (14)):

(14) $$m_{k}=\frac{1}{N_{k}}\sum_{i=1}^{N_{k}}x_{i},$$

where *C* is the number of classes, and *N_k_* is the number of the sensor responses in class *k*. LDA scores were also obtained using Equation (5); however, **x′** was a matrix of LDA scores. In this study, LDA was carried out on MATLAB R2018a (The MathWorks, Natick, MA, USA).

#### 2.4.3. Random Forests and ReliefF

To select appropriate sensors from the total of eight sensors in an array, random forest [32,33,34] and ReliefF [35,36,37] were carried out to obtain sensor weights against all data. A random forest repeatedly calculates the weight of each sensor using a randomly obtained part of the sensor responses and takes the average of the sensor weights. ReliefF selects one reference point from all the response values of each sensor and finds the number of data points of the same class (gas) and different classes of the *k* data points closest to the reference point, repeating this operation on all data to calculate weights. In this study, ReliefF was carried out with *k* values ranging from 1 to 10, but almost the same result was obtained. These results were used to examine which sensor of the eight sensors contributed most to the discrimination of the target gas, and to examine whether sensors can be thinned out. In this study, random forest and ReliefF were also carried out on MATLAB R2018a.

#### 2.4.4. k Nearest Neighbors (kNN)

The kNN algorithm was originally used as a method of data classification; it decides on classes by a majority vote of *k* data points with a short Euclidean distance to the unknown point [31]. In this study, PCA and LDA scores were regarded as unknown data, and kNN was performed to ascertain whether the class judged by the majority vote matched the score’s original class. This operation was carried out continuously until all scores could be regarded as reference scores. Such ratios were obtained for each class (2N, DA, PA, and AA). Scores from two target gases were defined as belonging to two classes each. In this study, *k* was set to 3, which produced the highest match ratios. The weighted averages of 2N, DA, PA, and AA were defined as the target for gas discrimination. The weighted averages of ratios were evaluated from scores without contamination gases (not included), scores with contamination gases (included), and all scores (all). From the ratios, we evaluated the effectiveness of sensor thinning. In this study, kNN was carried out using MATLAB R2018a.

## 3. Results and Discussion

### 3.1. Sensor Responses and Discrimination of Target Gases

**Figure 4:**
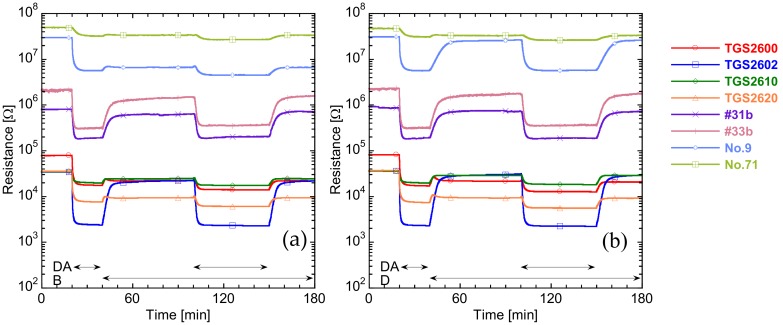
Dynamic resistance response of eight sensors to 5.4 ppm of diacetyl (DA) and 5.4 ppm of diacetyl with contaminant gases: (**a**) 2.7 ppm of butyl acetate (B) and (**b**) 1.0 ppm of *n*-decane (D). Graphs indicate flow periods of DA, B, and D.

Sensor responses *S* of eight sensors to the target gases were collected. Figure 4 shows the dynamic resistance response of all sensors to 5.4 ppm of diacetyl with and without contaminant gases, using 2.7 ppm of butyl acetate and 1.0 ppm of *n*-decane as an example. All eight sensors were n-type semiconductor gas sensors that decreased in resistance in response to VOCs. The first large resistance decrease at 20–40 min was a response to 5.4 ppm of diacetyl without contaminant gases. All sensors showed distinct sensor responses to diacetyl, especially TGS2602, #31b, #33b, and No. 9. At 40 min, diacetyl flow was stopped, and contaminant-gas flows were started, so that resistances of all sensors increased, but not to their original values. Resistance recovery was more affected by butyl acetate than *n*-decane because butyl acetate has an ester group and *n*-decane does not have oxygen atoms. Semiconductive gas sensors show similar resistive sensor responses to VOCs, having the same functional groups, and higher responses to oxyhydrocarbons, i.e., esters and alcohols, than aliphatic hydrocarbons [15]. The second large resistance decrease, at 100–150 min, indicated responses to 5.4 ppm of diacetyl with contaminant gases. Sensor resistance in 5.4 ppm of diacetyl (*R_g_*) with contaminant gases was slightly lower than that in 5.4 ppm of only diacetyl. However, since the decrease in resistance just before diacetyl flow began (*R_a_*) by contaminant gases was larger than the decrease in *R_g_*, so sensor-response values *R_a_*/*R_g_* decreased compared to the *R_a_*/*R_g_* without contaminant gases. Therefore, decreasing resistance responses to the mixed gas were not a simple addition of the resistance decreases for each gas. As a result, the sensor-response values were always affected by the contaminant gases. 

**Figure 5:**
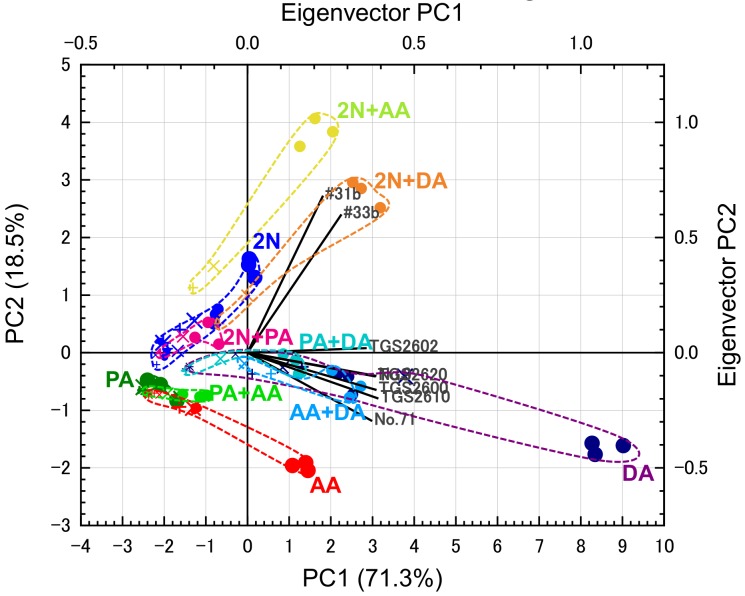
PCA scores and eigenvectors from eight sensors for one and two target gases (see Table 3 for types of plots).

Figure 5 shows the PCA scores and eigenvectors using the eight sensors. The cumulative variance of the first and second principal components (PCs) in Figure 5 was greater than 80%. To retain the originality of the data [38], the first two PCs are normally sufficient. We can see these PCA scores divided into three trends: first, PCA scores from 2N and gas combinations, including 2N (2N + AA, 2N + PA, and 2N + DA), were distributed in the first and second quadrant, and tended to monotonically increase; second, PCA scores from DA and gas combinations, including DA (PA + DA and AA + DA), were distributed in the third and fourth quadrant and tended to monotonically decrease; PCA scores from acids (AA, PA, and PA + AA) were also distributed in the third and fourth quadrant, and on the negative side of those from DA. The angle of the eigenvectors from #31b and #33b was similar to that of the area of 2N and gas combinations including 2N. The angle of the eigenvectors from TGS2600, 2620, 2610, No. 9, and No. 71 was also similar to that of the area of DA and acids. The eigenvectors from TGS2602 were between these eigenvectors.

The plots of PCA scores in PC1 and PC2 tended to move radially outward from around the co-ordinates (–2, –0.5) as the target-gas concentration increased. Diacetyl was used at a higher concentration (over 1 ppm) than the expected concentration in body odor. Since diacetyl has higher volatility than the other target gases, it is difficult to prepare low-concentration diacetyl gases under the same experiment conditions for the other target gases. However, PCA scores of low-concentration diacetyl gases can be expected to exist closer to the co-ordinates (–2, –0.5) than those of high-concentration diacetyl. When contaminations existed, the PCA scores moved in the direction of (–2, –0.5) compared to the target gas at the same concentration without contamination, and results were almost the same as when the target gas was at a low concentration without contamination because sensor response *R*_a_/*R*_g_ decreased in the presence of contaminants, as shown in Figure 4. However, since PCA scores in the presence of contaminants are generally within the area of each target gas, discrimination between the target gases was almost achieved.

**Figure 6:**
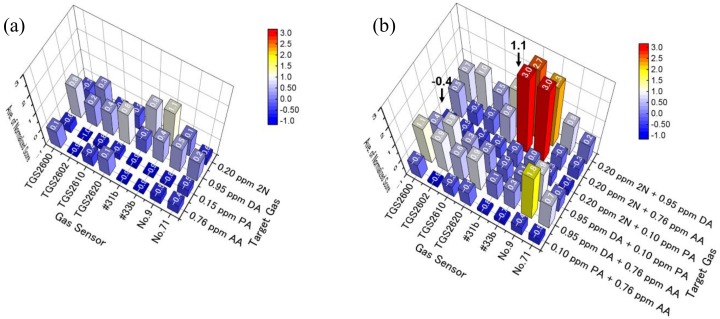
Average normalized scores of eight sensors to (**a**) one and (**b**) two target gases without contaminant gases.

Figure 6 shows the average normalized scores of all sensors to one and two target gases without contaminant gases. Normalized scores of #31b and #33b for 2N (Figure 6a) were larger than the other normalized scores. Furthermore, normalized scores for 2N + DA and 2N + AA were very large (Figure 6b). That is, PCA scores from 2N depended on #31b and #33b (Figure 5); #31b and #33b also contributed to the division of areas containing DA and acids. TGS2600, 2610, 2620, No. 9, and No. 71 showed high normalized scores for DA (Figure 6a) and double target gases including DA (Figure 6b), as well as intermediate normalized scores from AA and 2N (Figure 6a). Therefore, PCA scores from DA and acids depended on these sensors, and these sensors affected the area of 2N that was tilted from the eigenvectors of #31b and #33b (Figure 5). Since PA has very low saturated vapor pressure, we were unable to evaluate it at concentrations higher than 0.15 ppm. However, the pattern of PA was similar to the pattern in which average normalized scores of 0.76 ppm AA are generally small. Therefore, considering that semiconductor gas sensors show almost equivalent responses to the same group of gas species [15], we expected that the high-concentration PA would show PCA scores that were roughly equivalent to those of AA.

**Figure 7:**
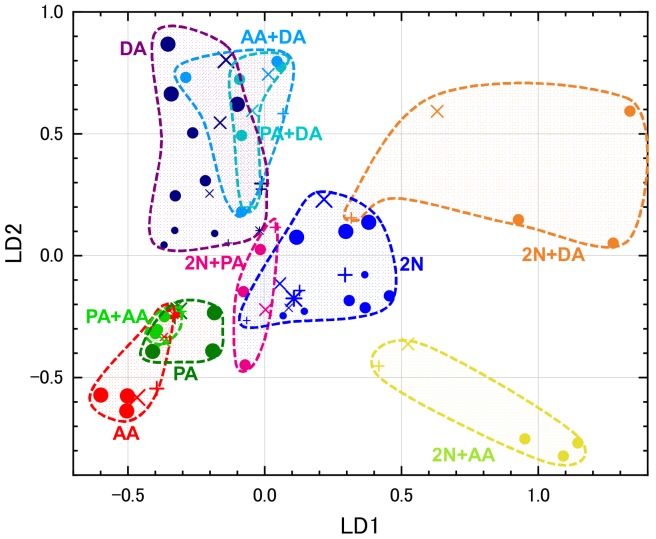
LDA scores from eight sensors for one and two target gases (see Table 3 for types of plots).

Figure 7 shows the LDA scores using eight sensors. In this study, LDA scores were classified by target-gas species without considering target-gas concentrations and contamination, so LDA scores tended to aggregate with the target-gas species. Variation in the plots due to changing concentration and the presence or absence of contaminant gas was also observed, but the direction of movement was not as clear as in PCA. The plots of each class in LDA seemed to be gathered compared to those of PCA. The area of each target gas in PCA is long and slender, so erroneous judgments seemed to be higher when using kNN. However, in the kNN evaluation shown in Table 4, there was hardly any difference in the match ratio between PCA and LDA. That is, although the shape of the area of each class differed, there was almost no difference in the condition of the number of plots in the vicinity. Regarding the influence of contaminant gas, as it surrounded the plots of each class in Figure 5 and Figure 7, it could be judged as roughly the same class by visual observation. In kNN, the match ratios in the presence of contaminant gases were lower than those in its absence, but the observed difference was not large. 

### 3.2. Selecting Appropriate Sensors for PCA and LDA

To select appropriate subsets of sensors, all sensor combinations had to be implemented with PCA and LDA. However, considering all combinations requires considerable calculation cost. In this study, random forest and ReliefF, as filter methods, were carried out to ascertain the weight of each sensor on the machine-learning results, indicating the effective feature quantities for classification and discrimination.

**Figure 8:**
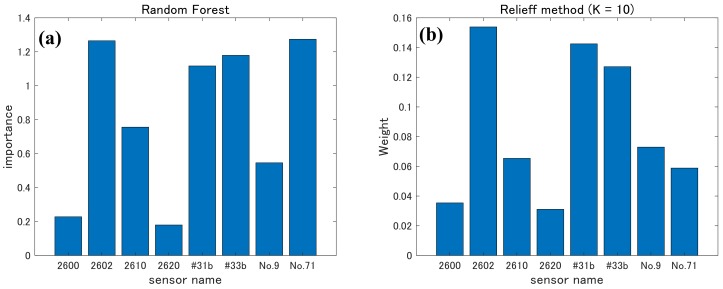
Weights given to results of each sensor obtained by (**a**) random forest and (**b**) ReliefF.

Figure 8 shows the weight given to the results of each sensor obtained by the random forest and ReliefF. Although results differed depending on the calculation method, the weights of TGS 2602, #31b, and #33b were large for both methods; the random forest also gave a high weight to No. 71. In both calculation methods, weights given to TGS 2600 and TGS 2620 were very small.

On the basis of random-forest and ReliefF results, a four-sensor set, namely, TGS2602, #31b, #33b, and No. 71, and a three-sensor set, namely, TGS2602, 2610, and #33b, were selected. Moreover, a five-sensor set with TGS2600, 2610, 2620, No. 9, and No. 71 that was not selected by ReliefF was also selected for comparison. PCA and LDA were carried out on the three combinations.

**Figure 9:**
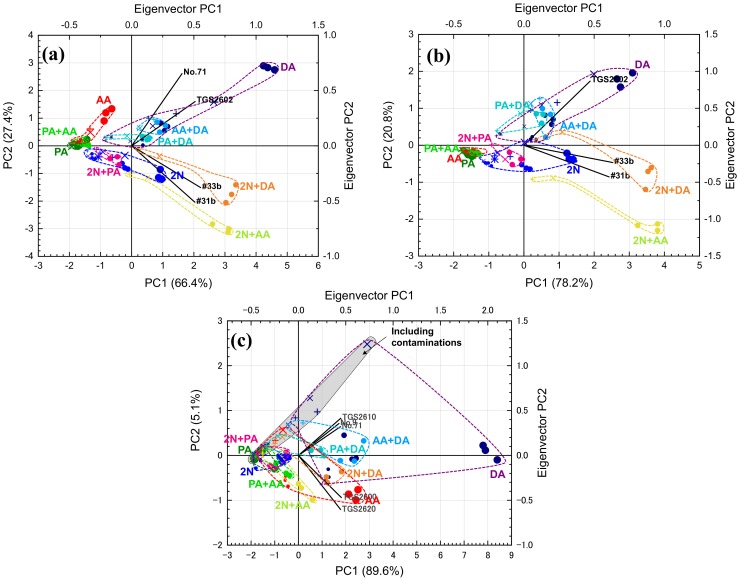
PCA scores and eigenvectors from (**a**) four sensors selected by random forest, (**b**) three sensors selected by ReliefF, and (**c**) five sensors not selected by ReliefF.

Figure 9 shows PCA scores from the four-, three-, and five-sensor sets. Figure 9a shows the PCA scores of the four sensors selected by the random forest. Despite the reversal of signs of the PC2 as compared with Figure 5, the separability of the PCA scores of each single gas and combination target seemed to be approximately equal. Since the overlap between the areas of 2N and 2N + DA was eliminated, their separability improved. Figure 9b shows the PCA scores from the three-sensor set selected by ReliefF. In this set, the area of the PCA score of AA became extremely narrow and it was difficult to judge its concentration. The three sensors selected by ReliefF did not include No. 71. By contrast, the four-sensor set selected by the random forest did, and the direction of the variation of the PCA score of AA was almost parallel to the eigenvector of No. 71. ReliefF excluded sensors that contain important information.

Figure 9c shows the PCA scores from the five-sensor set containing the sensors excluded by ReliefF. Compared to the results with eight sensors (Figure 5), the area of 2N decreased, and the area of DA and AA expanded, increasing their overlap with other areas. Moreover, scores from all target gases with contamination tended to be dense in the gray area in Figure 9c. That is, the scores were an arrangement in which the presence or absence of contamination was emphasized. Here, the amount of information regarding the separation of the target gases was reduced by excluding the three sensors judged to be useful by ReliefF for separating each target gas. Moreover, the cumulative variance of PC2 was very low (5.1%), meaning that there was almost no information in PC2, and the information possessed by the five sensors was similar. The five-sensor set is not preferable for discrimination.

**Figure 10:**
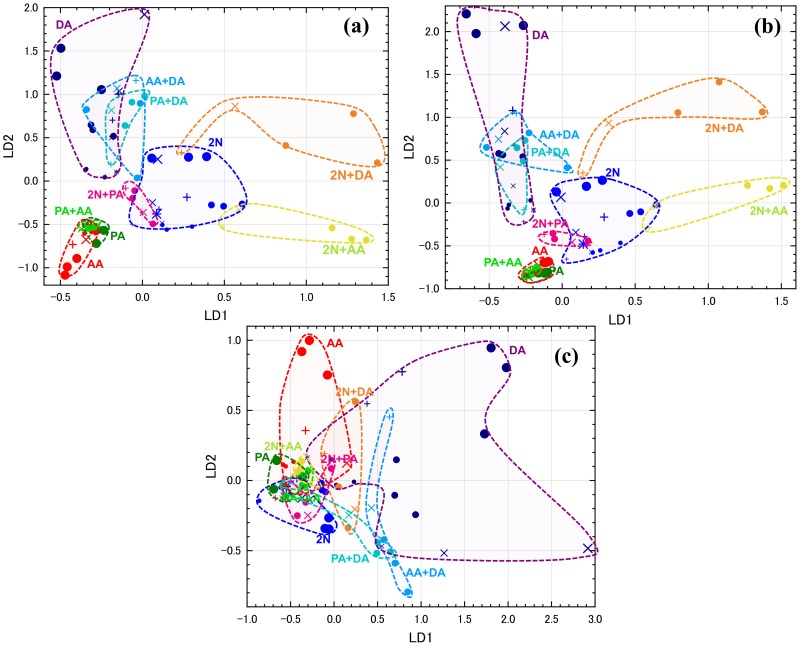
LDA scores from (**a**) four sensors selected by random forest, (**b**) three sensors selected by ReliefF, and (**c**) six sensors: TGS2602, TGS2610, #31b, #33b, No. 9, and No. 71.

Figure 10 shows LDA scores from the four-, three-, and five-sensor sets. From the four- and three-sensor sets (Figure 10a,b), no notable differences appeared, though minor variation occurred in each class area, especially the area of AA decreased in the three three-sensor set. In the five-sensor set (Figure 10c), discrimination deteriorated due to the increase in the area of DA and increasing compactness in other areas.

Table 5 shows the match ratios from PCA and LDA from the four-, three-, and five-sensor sets. The kNN values verified that the estimated discrimination by visual observation of PCA and LDA plots was almost correct. The match ratio from the four-sensor set using PCA was improved over that from the eight-sensor set (Table 4). The match ratio from the three-sensor set using PCA slightly deteriorated compared to that from all eight sensors. In the five-sensor set, the match ratio at all scores decreased, especially because the match ratio in cases including contamination greatly decreased. Although the match-ratio differences of LDA were smaller than those of PCA, match-ratio trends were almost the same as those of PCA. LDA is a supervised-learning method that finds axes that maximize the separation between multiple classes, so LDA scores tend to gather each class as compared to PCA.

On the basis of this result, it was possible to select sensors while preserving the discriminability of the target-gas type by preselecting weighted sensors via feature selection. Although this study did not discuss random-forest and ReliefF performance, it was important to select the optimal feature-selection method from the machine-learning result in order to select the optimal sensors.

## 4. Conclusions

Sensing analysis of target VOC gases with and without contamination was performed using an eight-sensor array, as well as four- (selected by random forest), three- (selected by ReliefF), and five-sensor (not selected by ReliefF) subsets of the array. PCA and LDA were nearly able to divide each class of target gases: the plots of the PCA scores tended to move radially outward from around the co-ordinates (–2, –0.5) as target-gas concentration increased, whereas LDA plots tended to aggregate with each target gas. Their scores to each target gas with contaminant gases were generally within the area of each target gas, so discrimination between each target gas was almost achieved. The match ratio from the four-sensor set using PCA improved as compared to that of the full sensor array, and the match ratio from the three-sensor set was almost as good, despite using only three sensors. The match ratio from the five-sensor set decreased because it used sensors determined by ReliefF to not contain much important information. Although differences in LDA match ratios were smaller than those of PCA, those of LDA showed the same trend as those of PCA. From this result, it is possible to reduce the required number of sensors while preserving the ability to discriminate between target-gas types by preselecting weighted sensors obtained using feature-selection techniques.

## Figures and Tables

**Table 1:** Details of four TGS sensors; two Pt, Pd, Au/SnO_2_; and two Zr-doped CeO_2_ sensors used in this study. VOCs, volatile organic compounds.

Sensor Name	Detected Compounds and Sensor Details
TGS 2600	H_2_ and alcohol (manufactured by Figaro)
TGS 2602	Alcohol, ammonia, VOC, and H_2_S (manufactured by Figaro)
TGS 2610	Liquefied petroleum gas (manufactured by Figaro)
TGS 2620	Alcohol and solvent vapors (manufactured by Figaro)
#31b	VOCs (Pt, Pd, Au/SnO_2_; film thickness: 4.6 μm)
#33b	VOCs (Pt, Pd, Au/SnO_2_; film thickness: 2.8 μm)
No. 9	Oxygen and VOCs (CeZr10; film thickness: 9 μm)
No. 71	Oxygen and VOCs (CeZr10/Al_2_O_3_/Pt-CeZr10; film thickness: 9/4/9 μm)

**Table 2:** Combinations of D-tubes employed in this study.

	VOCs	Concentration	Selection of D-tubes for Designated Concentration
Target gas	2N	0.20 ppm	D-04 × 1
		0.081 ppm	D-03 × 1
		0.021 ppm	D-01 × 1
	PA	0.15 ppm	D-05 × 3
		0.10 ppm	D-05 × 2
	DA	5.4 ppm	D-01 × 1
		1.6 ppm	D-001 × 1
		0.95 ppm	D-001* × 1
	AA	2.2 ppm	D-01 × 1
		0.76 ppm	D-001 × 1
Contaminant gas	B	2.7 ppm	D-02 × 1
	D	1.0 ppm	D-04 × 1

* A needle was inserted into a D-tube to decrease the diffusion amount.

**Table 3:** Plot shape and size of principal-component-analysis (PCA) and linear-discriminant-analysis (LDA) scores (Figures 5, 7, 9 and 10).

Target Gas (Class)	Concentration	Plot Color, Shape, and Size							
		Including Contaminant Gases							
		None		B (2.7 ppm)		D (1.0 ppm)		B (2.7 ppm) +D (1.0 ppm)	
2N	0.20 ppm	**●**	(large)	**+**	(large)	**×**	(large)	*	(large)
	0.081 ppm	**●**	(medium)	**+**	(medium)	**×**	(medium)	*	(medium)
	0.021 ppm	**●**	(small)	**+**	(small)	**×**	(small)	*	(small)
PA	0.15 ppm	**●**	(large)	**+**	(large)	**×**	(large)	*	(large)
	0.10 ppm	**●**	(small)	**+**	(small)	**×**	(small)	*	(small)
DA	5.4 ppm	**●**	(large)	**+**	(large)	**×**	(large)	*	(large)
	1.6 ppm	**●**	(medium)	**+**	(medium)	**×**	(medium)	*	(medium)
	0.95 ppm	**●**	(small)	**+**	(small)	**×**	(small)	*	(small)
AA	2.2 ppm	**●**	(large)	**+**	(large)	**×**	(large)	*	(large)
	0.76 ppm	**●**	(small)	**+**	(small)	**×**	(small)	*	(small)
2N + PA	0.20 ppm (2N) 0.10 ppm (PA)	**●**	(medium)	**+**	(medium)	**×**	(medium)	−	−
2N + DA	0.20 ppm (2N) 0.95 ppm (DA)	**●**	(medium)	**+**	(medium)	**×**	(medium)	−	−
2N + AA	0.20 ppm (2N) 0.76 ppm (AA)	**●**	(medium)	**+**	(medium)	**×**	(medium)	−	−
PA + DA	0.10 ppm (PA) 0.95 ppm (DA)	**●**	(medium)	**+**	(medium)	**×**	(medium)	−	−
PA + AA	0.10 ppm (PA) 0.76 ppm (AA)	**●**	(medium)	**+**	(medium)	**×**	(medium)	−	−
DA + AA	0.95 ppm (DA) 0.76 ppm (AA)	**●**	(medium)	**+**	(medium)	**×**	(medium)	−	−

**Table 4:** Weighted average of match ratios from PCA and LDA from eight sensors.

	**Match Ratio (%)**		
	**Contaminations**		
	**Not included**	**Included**	**All**
PCA from 8 sensors (Figure 5)	79	74	77
LDA from 8 sensors (Figure 7)	84	72	79

**Table 5:** Weighted averages of PCA and LDA match ratios from four-, three-, and five-sensor sets.

	**Match Ratio (%)**		
	**Contamination**		
	**Not included**	**Included**	**All**
PCA from 4 sensors (Figure 9a)	86	76	82
PCA from 3 sensors (Figure 9b)	78	72	75
PCA from 5 sensors (Figure 9c)	73	30	55
LDA from 4 sensors (Figure 10a)	81	74	78
LDA from 3 sensors (Figure 10b)	81	72	77
LDA from 5 sensors (Figure 10c)	68	54	62
